# The rs7404339 AA Genotype in *CDH5* Contributes to Increased Risks of Kawasaki Disease and Coronary Artery Lesions in a Southern Chinese Child Population

**DOI:** 10.3389/fcvm.2022.760982

**Published:** 2022-04-28

**Authors:** Yishuai Wang, Kun Lin, Linyuan Zhang, Yueling Lin, Hongyan Yu, Yufen Xu, Lanyan Fu, Lei Pi, Jinqing Li, Hanran Mai, Bing Wei, Zhiyong Jiang, Di Che, Xiaoqiong Gu

**Affiliations:** ^1^School of Medicine, South China University of Technology, Guangzhou, China; ^2^Department of Clinical Biological Resource Bank, Guangzhou Institute of Pediatrics, Guangzhou Women and Children’s Medical Center, Guangzhou, China; ^3^Department of Blood Transfusion and Clinical Lab, Guangzhou Institute of Pediatrics, Guangzhou Women and Children’s Medical Center, Guangzhou Medical University, Guangzhou, China; ^4^Department of Clinical Biological Resource Bank, Guangzhou Institute of Pediatrics, Guangzhou Women and Children’s Medical Center, Guangzhou Medical University, Guangzhou, China

**Keywords:** coronary artery lesion, cadherin-5, polymorphisms, Kawasaki disease (KD), southern Chinese child population

## Abstract

**Background:**

Kawasaki disease (KD) is an acute, self-limited febrile illness of unknown cause. And it predominantly affects children <5 years and the main complication is coronary artery lesion (CAL). Studies demonstrated that vascular endothelial cells (VECs) played a very important role in the CAL of KD. VE-cad encoded by *CDH5* may exert a relevant role in endothelial cell biology through controlling the cohesion of the intercellular junctions. The pathogenesis of KD remains unclear and genetic factors may increase susceptibility of KD. However, the relationship between *CDH5* polymorphisms and KD susceptibility has not been reported before. The present study is aimed at investigating whether the rs7404339 polymorphism in *CDH5* is associated with KD susceptibility and CAL in a southern Chinese child population.

**Methods and Results:**

We recruited 1,335 patients with KD and 1,669 healthy children. Each participant had supplied 2 mL of fresh blood in the clinical biologic bank at our hospital for other studies. Multiplex PCR is used to assess the genotypes of rs7404339 polymorphism in *CDH5*. According to the results, we found significant correlated relationship between rs7404339 polymorphism in *CDH5* and KD susceptibility [AA vs. GG: adjusted odds ratio (OR) = 1.43, 95% confidence interval (CI) = 1.00–2.05; *p* = 0.0493; recessive model: adjusted *OR* = 1.44, 95% *CI* = 1.01–2.06, *P* = 0.0431]. In further stratified analysis, we found that children younger than 60 months (adjusted *OR* = 1.46, 95% *CI* = 1.01–2.10; *p* = 0.0424) and male (adjusted *OR* = 1.70, 95% *CI* = 1.09–2.65; *p* = 0.0203) with the rs7404339 AA genotype in *CDH5* had a higher risk of KD than carriers of the GA/GG genotype. Furthermore, stratification analysis revealed that patients with the rs7404339 AA genotype exhibited the significantly higher onset risk for CAL than carriers of the GA/GG genotype (adjusted age and gender odds ratio = 1.56, 95% *CI* = 1.01–2.41; *P* = 0.0433).

**Conclusion:**

Our results showed that rs7404339 AA genotype in *CDH5* is significant associated with KD susceptibility. And children younger than 60 months and male with the rs7404339 AA genotype had a higher risk of KD than carriers with the GA/GG genotype. Furthermore, patients with the rs7404339 AA genotype exhibited a significantly higher risk of CAL complication than carriers of the GA/GG genotype.

## Introduction

Kawasaki disease (KD), an acute, self-limited systemic syndrome, is involved vasculitis along with fever. And it predominantly affects young children (age less than 5 years) (1, 2). KD is the most common acquired heart disease of children in developed countries these days and a severe medium-sized arteries vasculitis, exceptionally for coronary arteries (3). Approximately 20–25% of untreated patients had been developed to coronary artery lesion (CAL) or even coronary artery aneurysms (CAAs) (4, 5). In the acute stage, administration of a single high dose of intravenous immunoglobulin (IVIG) is an effective treatment to reduce the incidence of CAL. Unfortunately, about 3–5% of treated children still developed coronary artery abnormalities even CAAs (6). After IVIG treatment, the complication rate of KD is decreased to 5%. Epidemiological studies have reported that the incidence rates of KD were increasing in the area of Japan and Taiwan (7–9). This disease is an immune-mediated inflammatory response, which leaded to vascular endothelial injury (10). The etiology of KD may be related to infection, immune response, and gene factors. Recent studies have shown that genetic susceptibility played a more important role in KD than other factors. Genetic literature has reported that ITPR3 rs2229634, CASP3 rs72689236, and GRIN3A rs7849782 single nucleotide polymorphisms (SNPs) increase the risk of KD susceptibility and CAAs formation in KD patients in Taiwan (11–13). Dysregulation of the expression products of ITPR3, CASP3, and GRIN3A is associated with damaged vascular tissue and presents as subcutaneous edema, vascular injury, gap formation, and endothelial cell fenestration, which is the pathogenesis of this disease.

Human VE-cad protein is an endothelial-specific cadherin encoded by *CDH5* gene and located at intercellular junctions (14). VE-cad is the main component of endothelial cell adhesion and connection (15). It has been reported that its expression and phosphorylation can cause vascular endothelial dysfunction and increase microvascular permeability, which is closely related to a variety of diseases (16, 17). VE-cad consists of 780 amino acids, which are divided into three domains: extracellular, intracellular and transmembrane. It has also been reported that VE-cad can adhere to each other through extracellular regions (18). Phosphorylation of the tyrosine residue of VE-cad may lead to disruption of the cell-cell connection in specific microenvironments (19, 20). At the same time, skeletal rearrangement of vascular endothelial cells leads to the increase of endothelial cell contraction and endothelial space, further hindering the function of vascular endothelial barrier (21). Currently, endothelial barrier function of vascular endothelial cells is highly dependent on VE-cad complex connectivity under normal conditions (22). Therefore VE-cad is the key factor to regulate cell adhesion dynamics at endothelial junctions. VE-cad may play a role in endothelial cell biology by controlling the adhesion of intercellular junctions and maintaining the stability of endothelial cell junctions (23, 24). Diabetes and atherosclerosis are also associated with endothelial cell dysfunction and VE-cad, and elevated plasma VE-cad positive levels are associated with different levels of cardiovascular risk in patients with type 2 diabetes (25–28). Furthermore, Studies have reported that rs7499886 and rs1073584, two common intron variants of *CDH5* SNPs were significantly associated with central serous chorioretinopathy (29). As we all know, the primary focus of CSC is in the retinal pigment epithelium and choroidal capillaries, and the mechanism may be related to the increased permeability of choroidal capillaries, and abnormal hemodynamics or vascular regulation. We hypothesize that some structural changes of CDH5 protein may cause changes in vascular endothelial cells and lead to vasculitis in KD patients. Akira Narita reported that RS7189512 is associated with Autism Spectrum disorder, which is located between LINC00922 and *CDH5* (30). Our team have showed the associations of KD risk with SNPs, of IL-1β,miRNA-137 (31, 32). IL-1β and miRNA-137 encode pro-inflammatory cytokines that can induce endothelial cell apoptosis, which is the cause of endothelial damage in KD vessels and is associated with the development of the disease (33, 34). However, there is no literature which has previously been reported the relationships between rs7404339 polymorphism in *CDH5* and KD susceptibility. Thus, we carried out the present study.

## Materials and Methods

### Ethics Statement

The study is approved by the Medical Ethics Committee of Guangzhou Women and Children’s Medical Center (2014073009 and 2018052702). Informed written consent is obtained from the guardians of the patients and controls. The clinical trial registration number is ChiCTR-EOC-17013266 (seen in http://www.chictr.org.cn/showproj.aspx?proj=22637).

### Study Population

A total of 1,335 patients who were diagnosed as KD, and 1,669 healthy controls were recruited from January 2014 to December 2019. We diagnosed KD according to the American Heart Association guidelines (3, 4). The KD patients attended our hospital as outpatients with follow-ups and inpatients, and the healthy controls were children who came to our hospital for health examinations within the same time period and had no fever or other diseases. Each participant had supplied 2 mL of fresh blood. Total genomic DNA is extracted from 200 μL of each specimen which yielded an adequate amount for the genomic DNA analysis. We stored the rest of specimens in the clinical biological sample bank at our hospital for other studies. The present study is approved by the Guangzhou Women and Children Medical Center Ethics Committee, and the children and their families provided written informed consent.

### DNA Extraction and Genotyping

We had extracted Genomic DNA from 200 μL of blood collected from each participant using a TIANamp Blood DNA Kit (Tiangen, Beijing city, China) and we followed the specific procedures in the literature (35–37). The extracted DNA is placed in a −80°C refrigerator until use. The allele-specific probes were purchased from Applied Biosystems. TaqMan real-time polymerase chain reaction of the samples is performed in 384-well plate with an ABI Q6 instrument (Thermo Fisher Scientific, Waltham, MA, United States) to genotype rs7404339 polymorphism in *CDH5* (38, 39). For quality control, each 384-well plate contained eight samples without DNA but with the same amount of distilled water. Moreover, to ensure the quality and accuracy of the genotyping results, we randomly selected 10% of the samples for a repeat analysis, and the results were 100% concordant.

### Statistical Analysis

First, we used the chi-square test to evaluate the distributions of demographic variables and genotype frequencies in KD patients and controls. Then we used the chi-squared goodness-of-fit test to calculate Hardy-Weinberg equilibrium (HWE) for control samples. The association between the rs7404339 polymorphism in *CDH5* and KD susceptibility is as evaluated by calculating the odds ratio (OR) and the 95% confidence interval (CI). Then we performed an unconditional univariate logistic regression analysis. Adjusted ORs were calculated by multivariate analysis with adjustment for age and gender. We conducted all statistical analyses using SAS software (Version 9.1; SAS Institute, Cary, NC, United States), and *P* < 0.05 implied statistical significance.

## Results

### Clinical Characteristics of Patients With Kawasaki Disease

The clinical characteristics were summarized in Table 1. The clinical and demographic variables were from the recruited study population of 1,335 cases and 1,669 KD-free controls. The mean age of KD onset is 26.37 months. The KD group comprised 856 (64.12%) male patients and 479 (35.88%) female patients. We observed no significant differences between the KD patients and controls in terms of age (*P* = 0.7295) and gender (*P* = 0.0973). According to the American and Japan diagnostic guidelines, we defined CAL as Z-score is ≥ + 2.5 (3, 40). According to the coronary artery condition, the KD patients were divided into those with CAL (47.49%) and without CAL (NCAL) (52.51%).

**TABLE 1:** Characteristic distribution in Kawasaki disease (KD) cases and healthy controls.

	Cases (*n* = 1,335)		Controls (*n* = 1,669)		
Variables	No.	%	No.	%	*P* ^a^
Age range, month	1–151		1–168		
Mean ± SD	26.37 ± 22.22		33.37 ± 23.73		0.7295
≤60	1,241	92.96	1,546	92.63	
>60	94	7.04	123	7.37	
Gender					
Male	856	64.12	1,021	61.17	0.0973
Female	479	35.88	648	38.83	
**Coronary artery lesion**					
CAL	634	47.49			
NCAL	701	52.51			

*^a^Two-sided χ^2^ test for characteristic distributions in KD cases and controls. CAL, coronary artery lesion; NCAL, no coronary artery lesion.*

### Relationship Between the rs7404339 Polymorphism in *CDH5* and Kawasaki Disease Susceptibility

To explore the association between rs7404339 polymorphism in *CDH5* and KD susceptibility, we detected the genotype frequency distributions of KD cases and controls. As shown in Table 2, the controls satisfied the conditions for Hardy–Weinberg equilibrium (=0.6336). The genotype frequency distributions of the rs7404339 polymorphism in *CDH5* were 63.30% (GG), 31.46% (GA), and 5.24% (AA) in the KD group and 64.47% (GG), 31.88% (GA), and 3.65% (AA) in the controls. Our results showed that rs7404339 AA genotype in *CDH5* were correlated with KD susceptibility significantly [AA vs. GG: adjusted odds ratio (OR) = 1.43, 95% confidence interval (CI) = 1.00–2.05; *p* = 0.0493; recessive model: adjusted *OR* = 1.44, 95% *CI* = 1.01–2.06, *P* = 0.0431].

**TABLE 2:** Genotype frequency distributions of *CDH5* rs7404339 polymorphism in Kawasaki disease (KD) cases and controls.

genotype	Cases (*N* = 1,335)	Controls (*N* = 1,669)	P^a^	OR (95% CI)	P^b^	Adjusted OR (95% CI)	P^c^
***CDH5*/rs7404339 (HWE = 0.6336)**							
GG	845 (63.30)	1,076 (64.47)	0.1076	1.000		1.000	
GA	420 (31.46)	532 (31.88)		1.01 (0.86–1.18)	0.9473	0.98 (0.84–1.15)	0.8078
AA	70 (5.24)	61 (3.65)		1.46 (1.03–2.08)	**0.0362**	1.43 (1.00–2.05)	**0.0493**
				1.09 (0.96–1.23)	0.1881	1.07 (0.94–1.21)	0.3097
Dominant	490 (36.70)	593 (35.53)	0.5056	1.05 (0.91–1.22)	0.5052	1.03 (0.88–1.20)	0.7292
Recessive	1,265 (94.76)	1,608 (96.35)	**0.0348**	1.46 (1.03–2.07)	**0.0351**	1.44 (1.01–2.06)	**0.0431**

*^a^Two-sided χ^2^ test was used to determine differences of genotype distributions between KD cases and controls. ^b^Logistic regression analysis was used to evaluated the strength association between the CDH5 rs7404339 polymorphism and KD susceptibility. ^c^Adjusted for age and gender. The bold values mean the P values are statistically significant (P < 0.05).*

### Stratification Analysis of *CDH5* Gene Polymorphisms With Kawasaki Disease Susceptibility

We then further explored the association between *CDH5* gene polymorphisms and KD patients in stratified analyses considering age and gender (Table 3). We found that younger (≤60 months old) (*OR* = 1.47, 95% *CI* = 1.02–2.11, *P* = 0.0382, adjusted *OR* = 1.46, 95% *CI* = 1.01–2.09, *P* = 0.0424)and male (*OR* = 1.70, 95% *CI* = 1.10–2.63, *P* = 0.0179, adjusted *OR* = 1.70, 95% *CI* = 1.09–2.65, *P* = 0.0203) children with rs7404339 AA genotype were at significantly higher risk of KD than those with GG/GA genotypes. Furthermore, patients with the rs7404339 AA genotype exhibited significantly higher onset risk for CAL than carriers of the GA/GG genotypes (*OR* = 1.59, 95% *CI* = 1.04–2.42, *P* = 0.0321, adjusted *OR* = 1.56, 95% *CI* = 1.01–2.41, *P* = 0.0433).

**TABLE 3:** Stratification analysis of *CDH5* rs7404339 polymorphism in Kawasaki disease (KD) cases and controls.

	Patients/controls						
Variables	GA/GG	AA	P^a^	OR (95% CI)	P^b^	Adjusted OR (95% CI)	P^c^
**Age, months**							
≤60 months	1,175/1,489	66/57	**0.0379**	1.47 (1.02–2.11)	**0.0382**	1.46 (1.01–2.09)	**0.0424**
>60 months	90/119	4/4	0.6988	1.32 (0.32–5.43)	0.6984	1.31 (0.30–5.76)	0.7232
**Gender**							
Male	806/985	50/36	**0.0171**	1.70 (1.10–2.63)	**0.0179**	1.70 (1.09–2.65)	**0.0203**
Female	459/623	20/25	0.7883	1.09 (0.60–1.98)	0.7869	1.08 (0.59–1.97)	0.7997
**Coronary artery lesion**							
CAL	598/1,608	36/61	**0.0359**	1.59 (1.04–2.42)	**0.0321**	1.56 (1.01–2.41)	**0.0433**
NCAL	667/1,608	34/61	0.1833	1.34 (0.88–2.06)	0.1771	1.35 (0.87–2.07)	0.1769

*^a^Two-sided χ^2^ test was used to determine differences of genotype distributions between KD cases and controls based on different subgroups. ^b^Logistic regression analysis was used to evaluated the association between the rs7404339 AA polymorphism of CDH5 and KD susceptibility. ^c^Adjusted for age/gender. The bold values mean the P values are statistically significant (P < 0.05).*

## Discussion

Genetic susceptibility has become a concern for KD research, but no previous research has examined the association of the rs7404339 polymorphism in *CDH5* with KD. We analyzed the association between KD susceptibility and rs7404339 polymorphism in *CDH5* in our case–control investigation. Our study included 1,335 KD patients and 1,669 healthy controls. The results revealed a significant relationship between rs7404339 AA genotype in *CDH5* and KD susceptibility. Stratified analysis showed that *CDH5* rs7404339 AA genotype increased the risk of KD in men less than 60 months of age. In addition, subjects with rs7404339 AA genotype had a higher risk of developing CAL than subjects with GA/GG genotype. KD is also called mucocutaneous lymph node syndrome characterized by systemic vasculitis and it usually occurred in children younger than 5 years. The incidence rate of KD varies geographically. And it is more prevalent in the populations of Asia (41). KD has been extensively studied in terms of etiology, pathogenesis, treatment, prognosis, and intervention factors and causes of KD may be affected by viral or bacterial infections, autoimmune factors and genetic factors. While the pathogenesis of KD has not been clearly confirmed yet (42–44). Cytokines and inflammatory mediators interact with each other to develop the immune effect, which eventually lead to the persistence of VEC damage and aggravation. We have known that VEC damage is an important factor causing the coronary artery injury of KD (45). Children with KD have abnormal activation in immune system. The stimulated VEC in patients with KD can promote and release adhesion factors, and inflammatory cells adhere to the surface of VEC, leading to VEC damage (46). Endothelial dysfunction in patients with KD eventually leads to the formation of coronary artery aneurysms (47). The function of vascular endothelial barrier is largely dependent on cellular adhesion between endothelial cells (48). VE-cad is an endothelial specific adhesive protein located at the adherent junction and plays an important role in maintaining vascular integrity and endothelial barrier function (49). VE-cad mainly controls the opening and closing of the endothelial barrier in tissues, and its mediated contact regulates the formation of a selective semi-permeable barrier to control bidirectional migration between blood vessels and irrigated tissues (22). VE-cad, a Ca2 + dependent transmembrane adhesion glycoprotein, binds to P120 and β-catenin to form a stable junction complex that is essential for maintaining cell-cell adhesion (50). Stability of VE-cad-β-catenin complex blocks hyperpermeability of blood vessels and extravasation of leukocytes in inflamed cremaster, lung, and skin tissues (51). Therefore, we believe that the pathogenesis of KD may be related to endothelial injury caused by the disorder of VE-cad expression. Recently, it has been found that phosphorylation of VE-cad plays an important role in increased vascular leakage in diabetic retinopathy (52, 53). Studies showed blood pressure–associated sentinel SNP rs9337951 influence junctional VE-cad associated protein expression experimentally (54). It’s well-known that junctional VE-cad associated protein is a component of VE-cad-based cell-cell junctions in endothelial cells and contributes to atherosclerosis and endothelial cell dysfunction (55). The SNP of KIAA1462 gene has recently been reported to be associated with coronary artery disease risk, and the protein product of this gene is a novel component of cell-cell junctions (56). In addition, RS1412125 in HMGB1 gene was significantly correlated with the formation of CAL in KD patients (57). The polymorphism of c.212-37insC (rs3832879) in FGF23 gene may be related to the progression of CAL in KD children (58). Thus, it may indicate that the rs7404339 polymorphism in *CDH5* has a significant effect on coronary artery disease of KD by changing the expression of VE-cad.

We recruited 3,004 children (1,335 cases and 1,669 controls) to participate in our research. To the best of our knowledge, this study is the first investigation of the associations between rs7404339 polymorphism in *CDH5* and KD susceptibility in a southern Chinese child population. Compared with other previous studies, our study used a larger sample size and produced more statistically significant results. In this case control study, we also explored the association of this SNP with or without formation of CAL in a southern Chinese child population with KD. We found that the *CDH5* gene SNP is significantly associated with KD susceptibility in children less than age of 60 months and sex of male. And compared patients with GA/GG genotypes, carriers of the *CDH5* rs7404339 AA genotype had an increased risk of CAL (*P* = 0.0433). This result may be contributed to the fact that male and young children were more genetically susceptible to KD. Moreover, according to epidemiological studies, KD is an age- and gender-related disease which usually occurs in children with aged <5 years and sex of male (4, 59). However, the mechanism of these phenomenon is unclear and therefore our study has potential limitations that should be reviewed. Firstly, on account of the retrospective nature of the original study design, we had little detailed information about other factors, such as parental environmental exposures, dietary intakes. Secondly, we only conducted a case–control study to investigate the relationship between the rs7404339 polymorphism in *CDH5* and KD susceptibility, and we did not explore the expression level of *CDH5* in the peripheral blood or the potential mechanisms of action of the polymorphism. Thirdly, we only studied the southern Chinese child population, but we did not assess cases and controls from other regional groups.

In summary, the results of the present study are confirmed that the *CDH5* rs7404339 AA variant genotype is associated with significantly higher susceptibility of KD in a southern Chinese child population, especially among those younger than 5 years and male. Furthermore there is a significant relationship between rs7404339 AA genotype in *CDH5* and CAL susceptibility. While we need further investigate the mechanisms that rs7404339 polymorphism in *CDH5* affects KD susceptibility. As far as we know, phosphorylation and dephosphorylation of intercellular tyrosine residues in the VE-cad complex regulate VE-cad function (57). Wessel et al. found that Tyr685 and Tyr731 of VE-cad had significant selective regulation of vascular permeability or induction of leukocyte extravasation (58). This may provide a direction for studying the mechanism of *CDH5* expression change in KD patients. We have plans for functional research by using cell model or animal model to examine whether rs7404339 AA genotype causes dysfunction of VE-cad protein. Therefore, whether this SNP is related to the pathogenesis of KD vasculitis needs to be further studied. If we have known this gene’s function in KD, we can get valuable insights into its role in pathogenesis of this disease. However, to further characterize VE-cad and determine the mechanisms underlying its role in KD, we should perform multicenter studies involving practical experiments and also conduct molecular biological function study of the mechanism.

## Conclusion

Our study has shown a significant relationship between rs7404339 AA genotype in *CDH5* and KD risk in children younger than 60 months and male. In addition, we confirmed the rs7404339 AA genotype in *CDH5* increased risk of CAL in a southern Chinese child population.

## Data Availability Statement

The raw data supporting the conclusions of this article will be made available by the authors. Requests to access these datasets should be directed to the corresponding authors.

## Ethics Statement

The study was approved by the Medical Ethics Committee of Guangzhou Women and Children’s Medical Center (2014073009 and 2018052702). Informed written consent was obtained from the guardians of the patients and controls. The clinical trial registration number is ChiCTR-EOC-17013266 (seen in http://www.chictr.org.cn/showproj.aspx?proj=22637). Written informed consent to participate in this study was provided by the participants’ legal guardian/next of kin.

## Author Contributions

YW, KL, LZ, YL, LP, HM, JL, ZJ, and BW performed the research study and collected the samples and data. YX and HY analyzed the data. DC and XG designed the research study. YW and XG wrote the manuscript. YW and LF prepared all the tables. All authors contributed significantly to this work, reviewed the manuscript, and read and approved the manuscript.

## Conflict of Interest

The authors declare that the research was conducted in the absence of any commercial or financial relationships that could be construed as a potential conflict of interest.

## Publisher’s Note

All claims expressed in this article are solely those of the authors and do not necessarily represent those of their affiliated organizations, or those of the publisher, the editors and the reviewers. Any product that may be evaluated in this article, or claim that may be made by its manufacturer, is not guaranteed or endorsed by the publisher.
